# A guide in lentiviral vector production for hard-to-transfect cells, using cardiac-derived c-kit expressing cells as a model system

**DOI:** 10.1038/s41598-021-98657-7

**Published:** 2021-09-28

**Authors:** V. Kalidasan, Wai Hoe Ng, Oluwaseun Ayodeji Ishola, Nithya Ravichantar, Jun Jie Tan, Kumitaa Theva Das

**Affiliations:** 1 Infectomics Cluster, Advanced Medical and Dental Institute, Universiti Sains Malaysia, Kepala Batas, Malaysia; 2 Helmholtz Research Zentrum, Munich, Germany; 3 Regenerative Medicine Cluster, Advanced Medical and Dental Institute, Universiti Sains Malaysia, Kepala Batas, Malaysia

**Keywords:** Gene delivery, Gene therapy, Regenerative medicine, Stem-cell biotechnology

## Abstract

Gene therapy revolves around modifying genetic makeup by inserting foreign nucleic acids into targeted cells via gene delivery methods to treat a particular disease. While the genes targeted play a key role in gene therapy, the gene delivery system used is also of utmost importance as it determines the success of gene therapy. As primary cells and stem cells are often the target cells for gene therapy in clinical trials, the delivery system would need to be robust, and viral-based entries such as lentiviral vectors work best at transporting the transgene into the cells. However, even within lentiviral vectors, several parameters can affect the functionality of the delivery system. Using cardiac-derived c-kit expressing cells (CCs) as a model system, this study aims to optimize lentiviral production by investigating various experimental factors such as the generation of the lentiviral system, concentration method, and type of selection marker. Our findings showed that the 2nd generation system with pCMV-dR8.2 dvpr as the packaging plasmid produced a 7.3-fold higher yield of lentiviral production compared to psPAX2. Concentrating the virus with ultracentrifuge produced a higher viral titer at greater than 5 × 10^5^ infectious unit values/ml (IFU/ml). And lastly, the minimum inhibitory concentration (MIC) of puromycin selection marker was 10 μg/mL and 7 μg/mL for HEK293T and CCs, demonstrating the suitability of antibiotic selection for all cell types. This encouraging data can be extrapolated and applied to other difficult-to-transfect cells, such as different types of stem cells or primary cells.

## Introduction

Gene therapy is a molecular medicine approach that promises new treatments for numerous hereditary diseases^1,2^ by introducing genetic material into target cells to cure or slow down the progression of the disease. In the early stages of establishing gene therapy, most experiments were performed in vitro using lipofection or electroporation in cell lines that were easy-to-transfect^3^. However, cell lines are artificial model systems that do not necessarily reflect the biochemical status of primary cells or stem cells, which are often used for gene-based medicine. These cells are hard-to-transfect due to a lack of specific cell surface markers, have a more aggressive immune response, and exhibit a stringent membrane poration, inhibiting the entry of DNA-lipid complexes.

While non-viral vectors such as lipofection (i.e., Lipofectamine RNAiMAX Transfection Reagent)^4,5^, is low in cost and is non-pathogenic, it has limited transfection efficiency and has packaging constraints^6^. On the other hand, gene transfer with viral-based delivery systems is more successful and also addresses the gaps seen in non-viral vectors. Notably, lentiviral vectors can incorporate constructs up to 10 kB in size, transduce non-dividing cells and offer stable transgene expression as it can integrate into the human genome. Unlike adenoviral or adeno-associated vectors, there are rarely neutralizing antibodies against lentiviral vectors. Furthermore, the lentivirus has been modified to reduce biosafety risks, whereby the crucial genes are separated and packaged in several plasmids that form the viral glycoproteins^7^. Currently, there are four generations of lentiviral vectors which differ in the number of genetic constructs, the number of retained wild-type genes, and the number and type of heterologous elements that can affect vector titers and safety^8,9^.

This study utilizes cardiac-derived c-kit expressing cells (CCs) as a model for lentiviral-based gene delivery in hard-to-transfect cells. Since there is a broad potential use of gene therapy using CC, there is a need for an optimized protocol to transfect the cells using a lentiviral vector for an efficient, stable gene transfer. This study aimed to optimize the steps in lentivirus production, including the transfection method, the relative ratio of transfer, packaging and envelope plasmids, viral titering and purification, concentration method, and enriching for cells carrying the transgene. The experiments discussed in this paper also focused on lentiviral-based methods that would be applicable in any lab, without the need for state-of-the-art equipment to encourage the broad usage of gene therapy globally.

## Results

### Comparison of the efficiency of transfection with Lipofectamine 2000 or 3000

The efficiency was compared by transfecting pQBI-eGFP (a plasmid constituting GFP) into HEK293T cells with Lipofectamine 2000 or Lipofectamine 3000. GFP expression in the cells was monitored at 24-, 48- and 72-h post-transfection. Our observation revealed that the transfection efficiency using Lipofectamine 3000 was higher even at 24 h compared to cells transfected with Lipofectamine 2000. Successful transfection was observed with Lipofectamine 2000 only at 48 h. Our quantification using ImageJ showed that Lipofectamine 3000 had a 4.3-fold increase in transfection efficiency at 48 h than Lipofectamine 2000. As Lipofectamine 3000 performed better, it was used in all future experiments.

### Comparison of efficiency between 2nd and 3rd generation of lentiviral systems

**Figure 1:**
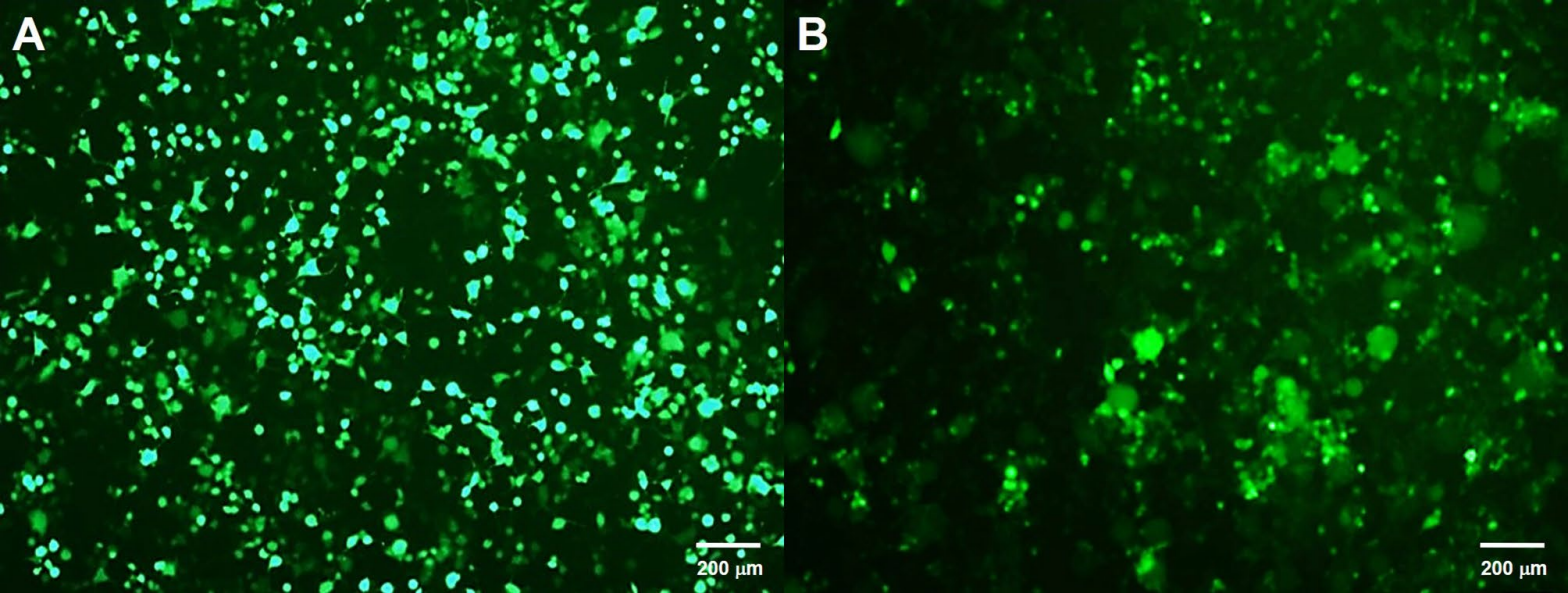
GFP expression in cells at 48-h post-transduction with the 2nd and 3rd lentiviral vectors. (**A**) Lentiviral vector 2A with pCMV-dR8.2 dvpr as the packaging plasmid produced the most lentivirus particle compared to 2B, and (**B**) Lentiviral vector 3B produced a smaller number of lentivirus particle compared to 3A.

In this experiment, the 1st generation lentiviral vector was omitted for safety reasons, while the 4th generation was not used as it was not commonly available. Only 2nd and 3rd lentiviral generations were used in this experiment based on their titer yield and transduction efficiency. Using ImageJ, quantification showed that the 2nd generation system with pCMV-dR8.2 dvpr as the packaging plasmid (2A) produced a 7.3-fold higher yield of lentiviral production compared to the 2nd generation system with psPAX2 packaging plasmid (2B). Meanwhile, 2A had a 1.7-fold and 2.6-fold higher viral yield compared to packaging plasmid 3A and 3B, respectively, the 3rd generation lentiviral plasmids. The GFP expression in cells at 48-h post-transduction with the 2A and 3B lentiviral vectors are shown in Fig. 1. The 2nd generation of lentiviral plasmids (2A) was used for all subsequent viral vector preparations due to higher titer, except in the proof-of-concept for puromycin selection, where 3B was used.

### Comparison of titering methods after lentiviral production

**Figure 2:**
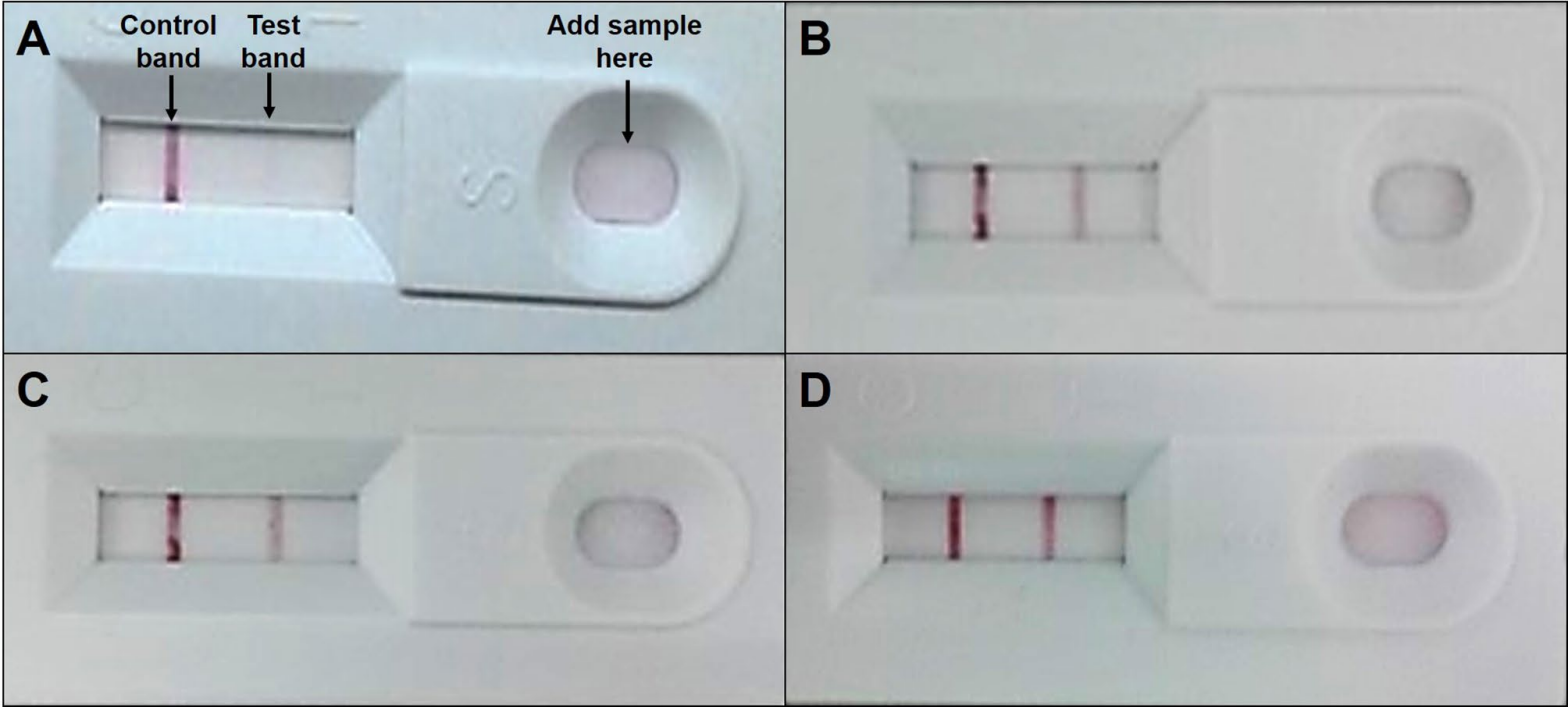
Lenti-X™ GoStix™ viral titration. Two bands on the stick indicate the presence of virus at greater than 5 × 10^5^ IFU/mL. (**A**) Negative control, (**B**) Lentiviral vector 2A, (**C**) Lentiviral vector 3A, and (**D**) Lentiviral vector 3B.

Lenti-X GoStix works by detecting p24, the viral core protein expressed during the early stages of infection. Two bands on the stick indicate the presence of p24 with a viral titer of greater than 5 × 10^5^ infectious unit values/ml (IFU/ml). For viral titration, 2B, which produced the lowest viral particles during lipid-based transfection, was omitted. The negative control and the lentivirus produced in 2A, 3A and 3B were titered using Lenti-X GoStix, as shown in Fig. 2.

### Comparison of the viral concentrating method using Lenti-X™ Concentrator and ultracentrifugation

**Figure 3:**
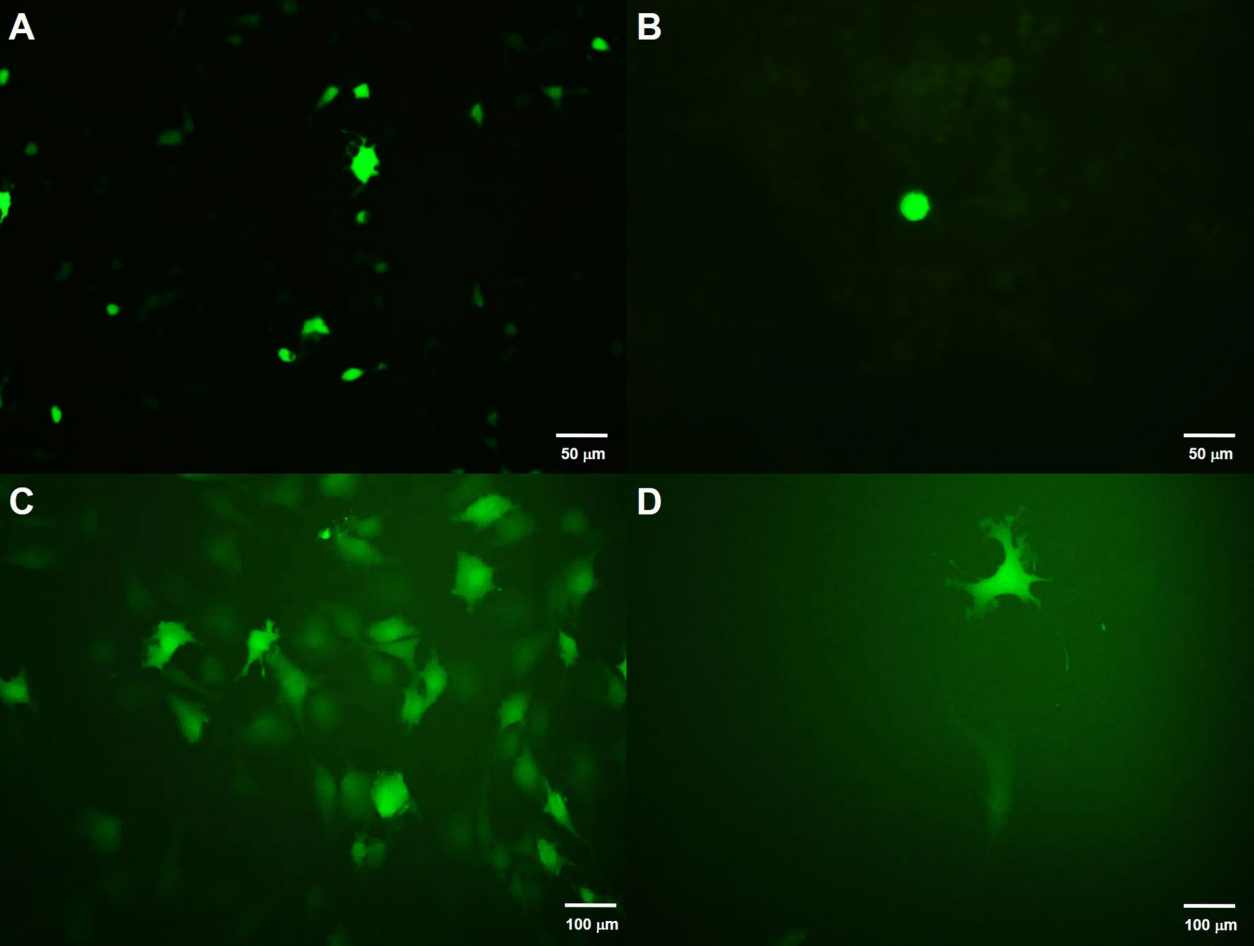
Lentiviral transduction CCs. (**A**) Successful transfection was observed in HEK293T cells using Lipofectamine 3000 after 24 h. (**B**) GFP-transduced CCs using lentivirus concentrated by Lenti-X™ Concentrator, (**C**) GFP-transduced CCs using lentivirus concentrated by ultracentrifugation, and (**D**) GFP-transduced CCs after passaging for one day.

For efficient delivery, the viral titer should be within 1 × 10^6^ to 1 × 10^10^ IFU/mL. In this study, the commercial reagents, Lenti-X Concentrator and ultracentrifugation method that can precipitate and concentrate the viral particles were used for comparison. Higher transduction efficiency in CCs was observed using viral concentrated from ultracentrifugation, compared to Lenti-X Concentrator, especially in CCs transduced with lentivirus 2A after 48 h, as shown in Fig. 3. An analysis of 4 replicates showed ~ 25% efficiency consistency, with a total of 746 cells indicating 25.2 + 1.2% of the transduced CCs expressed GFP.

### Comparison of puromycin selection

**Figure 4:**
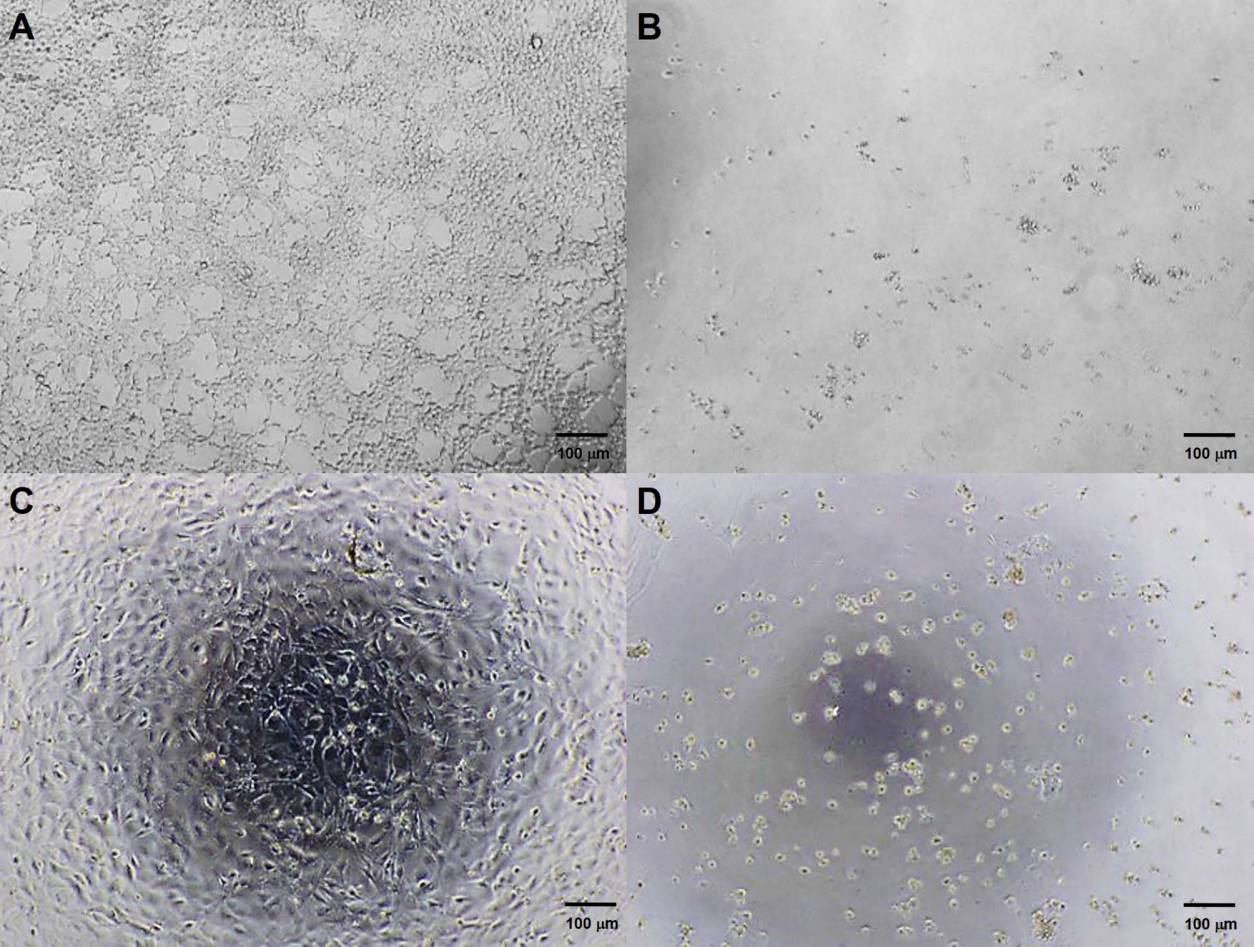
The minimum inhibitory concentration (MIC) of puromycin for the cells at day 5. (**A**) HEK293T with 0 µg/mL puromycin showed confluent adherent cells with a characteristic morphology, (**B**) HEK293T with 10 µg/mL puromycin showed 10 µg/ml dead cells, (**C**) CCs with 0 µg/mL puromycin showed confluent adherent cells with cell wall spikes, and (**D**) CCs with 7 µg/mL puromycin showed dead cells without spikes and floating.

As 2A did not contain any selectable markers, the 3B transgene with puromycin was used as the selection marker to confirm efficient delivery in HEK293T and CCs. The results showed the minimum inhibitory concentration (MIC) of puromycin was 10 μg/mL and 7 μg/mL for HEK293T and CCs, respectively. The morphology of the cells after the selection was compared between day 0 to day 5, as shown in Fig. 4.

## Discussion

**Figure 5:**
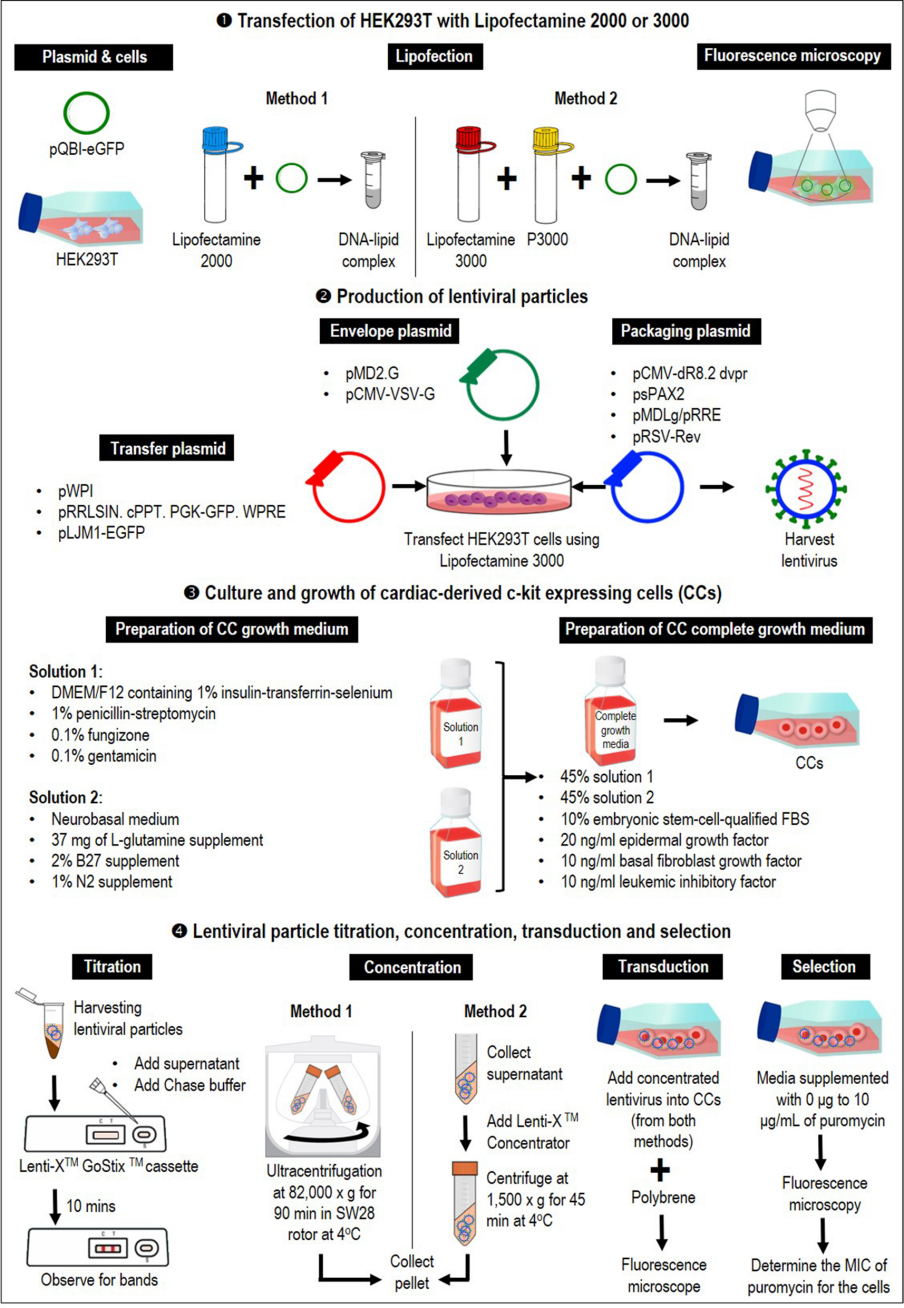
Diagrammatic workflow of the experiment. Preparation of complete growth media for CCs, optimizing different lipofection reagents, lentivirus production and the relative ratio of transfer, packaging and envelope plasmids, viral titering and purification, concentration, as well as selection method of cells carrying the transgene.

This study demonstrates a simplified lentiviral vector optimization that is cost and time effective and produces highly efficient transduction in CCs in transfection method, the relative ratio of transfer, packaging and envelope plasmids, viral titering and purification, concentration method, as well as selection method of cells carrying the transgene, as illustrated in Fig. 5. HEK293T cells act as host cells to assemble and generate viral particles before transduction was optimized.

In this study, lipofection was preferred for transportation of exogenous DNA by liposomes due to the properties and chemical composition of the cationic and neutral lipids. The transfection efficiency between Lipofectamine 2000 and 3000 was compared, and Lipofectamine 3000 worked for transfecting cells, in agreement with many past studies. Although transfection efficacy and toxicity of transfection reagents are highly cell-dependent^10^, Lipofectamine 2000, which has been widely used, displayed high toxicity in most cell types tested. In contrast, Lipofectamine 3000 demonstrated a high transfection efficacy in most cells, including in HEK293 and human umbilical vein endothelial cells (HUVECs) and showed stable co-transfection with multiple linear DNA^11^. Overall, in agreement with past studies, we found Lipofectamine 3000 to be an efficient and reproducible transfection reagent for biologically relevant cell models compared to other lipid-based reagents (i.e., Lipofectamine 2000 and FuGENE reagents).

After optimizing the transfection reagent, the transfection efficiency, the titer yield of 2nd and 3rd generation lentiviral vectors and their transduction efficiency on CCs were investigated. The packaging vector of the 2nd generation of lentivirus contained Gag, Pol, Tat and Rev in a single plasmid, while in the 3rd generation, the packaging vector was divided into two different plasmids (i.e., one encoding Gag and Pol and another encoding Rev) to improve the safety of the system^12,13^. This study showed that the 2nd generation system with pCMV-dR8.2 dvpr as the packaging plasmid (2A) had an increased efficiency and higher reproducibility compared to the 3rd generation lentivirus vector (3B). Similarly, a study reported that the 2nd generation lentivirus vectors produced approximately fifty times more total yield than 3rd generation vectors. The latter also did not have any beneficial effect on the ability of the viral vector to transduce primary human CD45RA^+^ T cells^14^. Although deemed to be safe, the 3rd generation of lentiviral necessitates the successful co-transfection of four different plasmids (including the transfer vector and envelope) to produce functional particles^15,16^. Due to the high number of plasmid requirements, lower viral titers and reduced transduction efficiency were observed in this study. Overall, the plasmid ratios and amounts were optimized based on previously published papers to ensure successful transfection. Despite the differences in promoters in the transfer vectors, different plasmids were used based on the recommended combinations from groups that had previously worked with these vectors. This approach also enabled the efficacy of publicly available lentiviral plasmids to be gauged, reducing the need to only purchase specific commercialized plasmids, which are typically less affordable.

The next step after selecting the most suitable lentiviral vector is to ensure an adequate number of viral particles for transduction. Viral titering is performed to analyze the quality of lentiviral vector production, gene transfer efficiency, and the level of therapeutic gene expression^17^. There are various methods to titer the virus, such as p24 ELISA, limiting dilution, quantitative PCR, and reporter genes expression^18–20^. Although these methods are efficient, they can be tedious and time-consuming. Thus, a commercially available kit, Lenti-X GoStix, was used in this study, which was quick and produced results within ten minutes. The virus particles from 2A, 3A and 3B expressed two bands when titered with Lenti-X GoStix, implying that the virus was successfully produced, in agreement with the data seen with fluorescent microscopy. Previous studies have demonstrated the usage of semi-quantitative assessment of viral titer using Lenti-X GoStix for transducing cardiomyocytes. Furthermore, the GoStix Value (GV; equivalent to ng/ml p24) has been proven to be correlated to infectious titer when using traditional p24 ELISAs, or qRT-PCR assay as a suitable substitute for other methods of functional titration. This technique requires minimal technical skills, it can easily be adopted even by beginners in virus-related work.

To improve the viral transduction efficiency in subsequent testing, virus can be purified and concentrated using several methods, such as ultracentrifugation or ultrafiltration^14^. In this study, we compared ultracentrifugation and a commercially available kit, Lenti-X Concentrator. Lenti-X Concentrator is simple, scalable to any volume and concentrates virus in less than an hour. On the other hand, ultracentrifugation, a more established method, requires a longer time to concentrate the virus (90 min). Our study showed higher transduction efficiency in CCs using virus concentrated from ultracentrifugation compared to Lenti-X Concentrator. Numerous studies have shown that the concentration of the culture supernatants achieved an increase in the viral titers by ultracentrifugation and was the preferred method since there is no evidence of toxicity by lentivirus vectors prepared using this technique^19,21^. Newer methods related to ultracentrifugation have recently been established, such as sucrose gradient centrifugation with a relatively low speed (≤ 10,000 g), which robustly produces a high-titer virus (up to 2 × 10^8^ TU/ml)^22^. This method is an efficient and easy-to-handle protocol for high-titer lentivirus purification and surpasses the instrumental barrier for routine laboratory operations.

Following transduction, not all target cells might receive the transgene. Cell enrichment can be conducted to select successfully transduced cells containing the transgene from a mixture of cells. Different methods can be used to determine efficient transgene delivery^23^, such as drug-resistant genes, intracellular enzymes, and fluorescent proteins (GFP). As the viability of cells can be affected by cell sorting, this study used transgene with puromycin as a selection marker that enabled an effective positive selection of cells expressing the puromycin-N-acetyltransferase (*pac*) gene. Similar to many of the methods used in this study, substituting cell sorting during cell enrichment with this method would eliminate the need for high-end equipment. Our study showed at day 0, the CCs showed adherent confluent cells with wall spikes, which later degenerated without spikes on day 5 (at 8 µg/mL of puromycin), proving that this simple method can be completed over a short time span, in less than a week.

**Figure 6:**
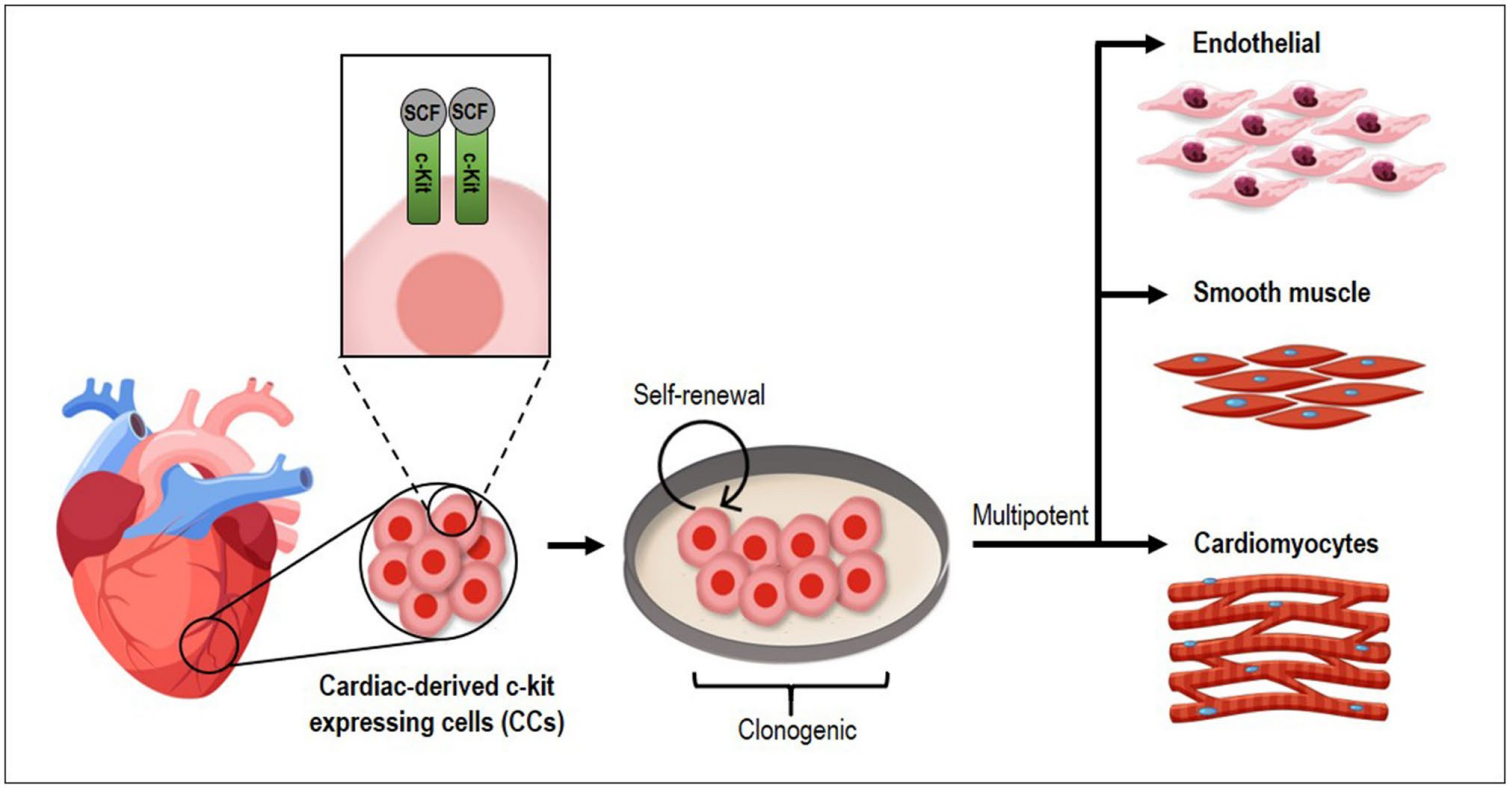
Cardiac-derived c-kit expressing cells (CCs) are self-renewal, clonogenic, and multipotent give rise to cardiomyocytes, smooth muscle, and endothelial cells. c-Kit is a type III receptor tyrosine kinase (RTK) transmembrane receptor involved in multiple intracellular signalling and considered a ligand for stem cell factor (SCF).

Overall, we provided a comprehensive overview of lentiviral vector production for hard-to-transfect cells, using CCs as a model system. Cells that are c-kit positive have properties of cardiac stem cells, including the capability to self-renew, are clonogenic, multipotent, and give rise to myocytes, smooth muscle, and endothelial cells^24,25^, as shown in Fig. 6. c-Kit is a type III receptor tyrosine kinase (RTK) transmembrane receptor involved in multiple intracellular signalling and considered as a ligand for stem cell factor (SCF), which participates in vital functions of the mammalian body and holds immense potential for therapeutic purposes^26–28^.

**Figure 7:**
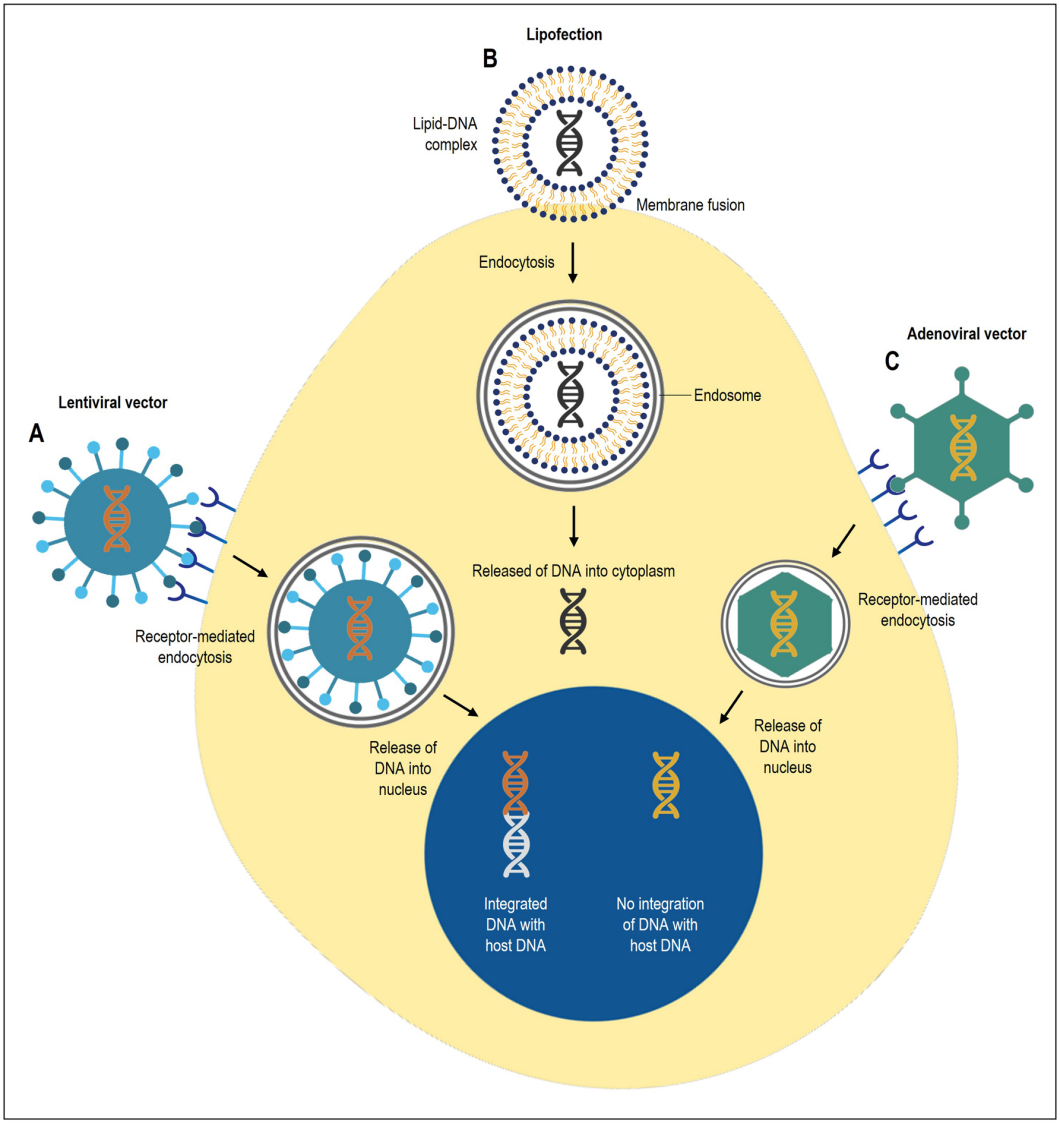
Comparison between non-viral and viral vectors gene delivery systems. (**A**) Lentiviral vector has up to 10 kb packaging capacity with high transduction efficiency. It is a stable expression system that integrates with host DNA and allows for long-term expression. (**B**) Non-viral vectors such as lipofection complexes DNA with liposomes and protects DNA from enzymatic degradation once released into the cell cytoplasm. It is a simple method with high packaging capacity and low immunogenicity. It is a transient expression system. (**C**) Adenoviral vector has up to 36 kb packaging capacity with high transduction efficiency. It is a transient expression system and immunogenic, which elicits a strong immune response.

Current transgene delivery utilizing adenoviral or adeno-associated vectors that directly target adult cardiomyocytes are still under investigation for cardiac gene therapy^29–33^. However, this approach is limited by the ability of cardiomyocyte proliferation^34,35^. Thus, genetic materials cannot be passed down to sustain therapeutic benefits. Adenoviral vector has up to 36 kb packaging capacity with high transduction efficiency^36–40^. While it has a large capacity, it is a transient expression system and immunogenic, which elicits a strong immune response, making it an unsuitable delivery method for gene therapy. On the other hand, the adeno-associated viral system is affected by neutralizing antibodies, which will significantly reduce the transduction efficiency^41–43^. In contrast, lentiviral systems allow stable genome integration into the host cells and have the added advantage of high transduction efficiency in hard-to-transfect cells and cardiovascular gene therapy^6,44^. The balance of important features in lentiviral systems makes it a suitable system for long-term gene delivery. The comparison between lipofection, lentiviral and adenoviral vectors gene delivery systems is illustrated in Fig. 7.

Cardiac c-kit cells remain interesting regenerative cells for repairing damaged hearts. Despite the ongoing debates over its cardiomyocyte commitment^45^, nonetheless, evidence has supported the function of cardiac c-kit cells in salvaging damaged hearts^46,47^. Labelling purified rat cardiac cells with reporter genes was previously achieved by employing a closed commercial lentivirus system^46^ or by direct isolation from GFP-expressing transgenic rat hearts^48^. Whilst the underlying mechanism and the function of the cells would need to be further explored, this manuscript offers the most effective and precise molecular tracking method to unveil the cell fate and functions of such cells in a more complex setting such as coculture, three-dimensional tissue engineering and the heart in vivo*.*

We acknowledge that the limitation of this study is that there are several other options for specific stages of lentivirus production that was not investigated in this study. However, the primary purpose of this study was to identify steps that can be easily replicated in most laboratories when working with hard-to-transfect cells without having to purchase any additional equipment or incurring extra costs. Using CCs as a model system for lentiviral vector production, this encouraging data can be extrapolated and applied to other difficult-to-transfect cells, such as different types of stem cells or primary cells. In the future, we plan to compare the efficacy of fourth-generation lentiviral plasmids to take into account safety when establishing delivery systems for gene therapy.

## Methods

### Transfection of HEK293T with Lipofectamine 2000 or 3000

6.25 × 10^5^ human embryonic kidney (HEK293T) were seeded in a T75 cm^2^ flask containing Dulbecco’s Modified Eagles Medium (DMEM) supplemented with 10% FBS and 1% penicillin/ streptomycin at 37 °C and 5% CO_2_ (All the above were purchased from Gibco®, Invitrogen Life Technologies Co., CA, USA). When HEK293Ts were at 90% confluency, transfection was done using Lipofectamine 2000 and Lipofectamine 3000, respectively, according to manufacturers’ protocol (Thermo Fisher Scientific, MA, USA). The transfection reagent for each Lipofectamine product is as follows: 2.5 µg pQBI-eGFP: Lipofectamine 2000 in a 1:1 volume ratio, and 2.5 µg pQBI-eGFP, 3.75 µL Lipofectamine 3000 and 5 µL p3000. Cells expressing GFP expression under both transfection conditions were monitored under a fluorescence microscope at 10 × at days 1, 2 and 3 post-transfections to determine the efficiency of transient transfection in HEK293Ts.

### Lentiviral plasmids

The following lentiviral plasmids were used for this experiment: pWPI was a gift from Didier Trono (Addgene plasmid # 12254; http://n2t.net/addgene:12254; RRID:Addgene_12254), pCMV-dR8.2 dvpr was a gift from Bob Weinberg (Addgene plasmid # 8455; http://n2t.net/addgene:8455; RRID:Addgene_8455), pMD2.G was a gift from Didier Trono (Addgene plasmid # 12259; http://n2t.net/addgene:12259; RRID:Addgene_12259), pRRLSIN.cPPT.PGK-GFP.WPRE was a gift from Didier Trono (Addgene plasmid # 12252; http://n2t.net/addgene:12252; RRID:Addgene_12252), psPAX2 was a gift from Didier Trono (Addgene plasmid # 12260; http://n2t.net/addgene:12260; RRID:Addgene_12260), pLJM1-EGFP was a gift from David Sabatini (Addgene plasmid # 19319; http://n2t.net/addgene:19319; RRID:Addgene_19319), pMDLg/pRRE was a gift from Didier Trono (Addgene plasmid # 12251; http://n2t.net/addgene:12251; RRID:Addgene_12251), pRSV-Rev was a gift from Didier Trono (Addgene plasmid # 12253; http://n2t.net/addgene:12253 ; RRID:Addgene_12253), and pCMV-VSV-G was a gift from Bob Weinberg (Addgene plasmid # 8454; http://n2t.net/addgene:8454; RRID:Addgene_8454).

### Production of lentiviral particles using 2nd and 3rd generation lentiviral plasmids

The HEK293Ts were grown using the same cell density, medium and culture conditions, as discussed in Sect. 1. After 24 h, at 90% cell confluency, cells were transfected with Lipofectamine 3000 with 2nd generation lentiviral plasmids or 3rd generation lentiviral plasmids in the ratios shown in Table 1 for a total of 8 µg. 24 h post-transfection, the media was replaced with complete media. The supernatant was harvested 48 h post-transfection to verify the presence of the virus.

**Table 1:** Plasmids and ratios used to produce lentiviral vectors in this study.

Generation	Name	Addgene number	Plasmid	Ratio	Reference
2nd	2A	12,254	Transfer plasmid: pWPI	5	Trono Lab Constitutive Lentiviral Plasmids (unpublished)
		8455	Packaging plasmid: pCMV-dR8.2 dvpr	2	Stewart et al. (2003)
		12,259	Envelope plasmid: pMD2.G	1	Trono Lab Constitutive Lentiviral Plasmids (unpublished)
2nd	2B	12,252	Transfer plasmid: pRRLSIN. cPPT. PGK-GFP. WPRE	4	Trono Lab Constitutive Lentiviral Plasmids (unpublished)
		12,260	Packaging plasmid: psPAX2	3	Trono Lab Constitutive Lentiviral Plasmids (unpublished)
		12,259	Envelope plasmid: pMD2.G	1	Trono Lab Constitutive Lentiviral Plasmids (unpublished)
3rd	3A	19,319	Transfer plasmid: pLJM1-EGFP	4	Sancak et al. (2008)
		12,251	Packaging plasmid: pMDLg/pRRE	2	Dull et al. (1998)
		12,253	Packaging plasmid: pRSV-Rev	1	Dull et al. (1998)
		12,259	Envelope plasmid: pMD2.G	1	Trono Lab Constitutive Lentiviral Plasmids (unpublished)
3rd	3B	19,319	Transfer plasmid: pLJM1-EGFP	4	Sancak et al. (2008)
		12,251	Packaging plasmid: pMDLg/pRRE	2	Dull et al. (1998)
		12,253	Packaging plasmid: pRSV-Rev	1	Dull et al. (1998)
		8454	Envelope plasmid: pCMV-VSV-G	1	Stewart et al. (2003)

### Lentiviral particle titration

Forty-eight hours post-transfection, viral titer was confirmed by Lenti-X GoStix, an instant lentiviral titer test, according to manufacturer’s protocol (Takara Bio USA, Inc. CA, USA). Lentiviral particles from the supernatant were harvested by centrifugation at 500 × *g* for 3 min to separate HEK 293 T from the lentivirus and filtered through a 0.45 µm pore sized filter membrane to remove the cell debris. 20 μL of the lentiviral supernatant, followed by three drops of Chase Buffer, was applied to the sample well of the GoStix cassette and observed for control and test bands to indicate the presence of lentiviral particles (1 × 10^5^ particles).

The Lenti-X GoStix is designed to measure the amount of p24 capsid protein present in lentiviral supernatants. The intensity of the test band on the cassette increases with the amount of p24 protein in the lentiviral supernatant, which correlates with increasing infectious units (IFU).

### Lentiviral particle concentration

Once the presence of lentivirus was confirmed, the supernatant was divided into two and concentrated either with ultracentrifugation or Lenti-X Concentrator according to manufacturer’s protocol (Takara Bio USA, Inc. CA, USA). For ultracentrifugation, viral particles were concentrated at 82,000 × *g* for 90 min in an SW28 rotor at 4 °C. For Lenti-X Concentrator, viral supernatant was centrifuged at 500 × *g* for 10 min and filtered through a 0.45 μm filter to remove debris. Three volumes of supernatant were combined with one volume of Lenti-X™ Concentrator and incubated at 4 °C for 30 min. Samples were centrifuged at 1,500 × *g* for 45 min at 4 °C. For both ultracentrifugation and Lenti-X Concentrator, the supernatant was removed, the pellet was resuspended in 400 μL complete media and stored at − 80 °C for long term storage.

### Transduction of CCs with concentrated lentiviral particles

6.25 × 10^5^ of CC cells were seeded in a 6-well plate and cultured in the CC growth medium. CC growth medium was made up of two solutions: solution 1 comprised of DMEM/F12 containing 1% (v/v) insulin-transferrin-selenium, 1% (v/v) penicillin–streptomycin, 0.1% (v/v) fungizone, and 0.1% (v/v) gentamicin, while solution 2 comprised of neurobasal medium supplemented with 37 mg of L-glutamine, 2% (v/v) B27 supplement, and 1% (v/v) N2 supplement. The complete growth medium (CGM) was prepared by mixing the solutions in the ratio of 45% solution 1, 45% solution 2 and 10% (v/v) embryonic stem cell-qualified FBS (All of the above were purchased from Gibco®, Invitrogen Life Technologies Co., CA, USA). Finally, CGM was supplemented with 20 ng/ml epidermal growth factor, 10 ng/ml basal fibroblast growth factor and 10 ng/ml leukaemia inhibitory factor (All growth factors were purchased from Peprotech, Rocky Hill, NG, USA). Cells were incubated at 37 °C and 5% CO_2_ overnight. After 24 h, 100 µL of lentivirus concentrated with ultracentrifugation and Lenti-X™ Concentrator were used to transduce the cells. Polybrene was added to the media for a final concentration of 8 μg/mL. At 48 h post-transduction, media was replaced with fresh media and transduction efficiency was determined using fluorescent microscopy.

### Puromycin selection for successfully transduced CCs

2.0 × 10^5^ HEK293T and CCs were plated in a 6-well plate with media supplemented with 0 μg to 10 μg/mL of puromycin for nine days. The medium was changed to fresh media after 72 h and observed under the microscope every day to determine the MIC of puromycin for the cells.
